# Exogenous spermine alleviates the negative effects of combined salinity and paraquat in tomato plants by decreasing stress-induced oxidative damage

**DOI:** 10.3389/fpls.2023.1193207

**Published:** 2023-05-09

**Authors:** Lidia S. Pascual, María F. López-Climent, Clara Segarra-Medina, Aurelio Gómez-Cadenas, Sara I. Zandalinas

**Affiliations:** Department of Biology, Biochemistry and Environmental Sciences, University Jaume I, Castellón, Spain

**Keywords:** spermine, stress combination, ROS, tomato, polyamine, climate change, salinity, herbicide

## Abstract

Plants are frequently exposed to different combinations of soil constraints including salinity and different herbicides. These abiotic conditions negatively affect photosynthesis, growth and plant development resulting in limitations in agriculture production. To respond to these conditions, plants accumulate different metabolites that restore cellular homeostasis and are key for stress acclimation processes. In this work, we analyzed the role of exogenous spermine (Spm), a polyamine involved in plant tolerance to abiotic stress, in tomato responses to the combination of salinity (S) and the herbicide paraquat (PQ). Our findings showed that application of Spm reduced leaf damage and enhanced survival, growth, photosystem II function and photosynthetic rate of tomato plants subjected to the combination of S and PQ. In addition, we revealed that exogenous Spm reduced H_2_O_2_ and malondialdehyde (MDA) accumulation in plants subjected to S+PQ, suggesting that the role of exogenous Spm in alleviating the negative effects of this stress combination could be attributed to a decrease in stress-induced oxidative damage in tomato plants. Taken together, our results identify a key role for Spm in improving plant tolerance to combined stress.

## 1 Introduction

Plants growing in nature normally experience different combinations of climate threats, including heat, drought, sudden flooding, or cold snaps, among others, that negatively affect their productivity, growth and development (Yadav et al., 2020; Masson-Delmotte et al., 2021; Pascual et al., 2022). In addition, the devastating effect of human activities on soils results in poor soil quality characterized by increased amounts of salinity, herbicides, microplastics, heavy metals, nutrition deficiencies or changes in pH and microbial diversity that pose a serious challenge for agricultural production (Rillig et al., 2019; Zandalinas et al., 2021b; Pascual et al., 2022; Sinha et al., 2022; Speißer et al., 2022). Therefore, plants are constantly subjected to different combinations of two or more of these conditions (Zandalinas et al., 2021a; Zandalinas and Mittler, 2022). The excessive use of herbicides along with increased salt toxicity are among the major soil-associated abiotic stresses that diminish agricultural production all over the world (Pitman and Läuchli, 2002). Paraquat (PQ; also known as methyl viologen; 1,1′-dimethyl-4,4′-bipyridinium dichloride) is a reactive oxygen species (ROS)-producing, rapidly acting and non-selective herbicide (Hawkes, 2014). In turn, excess salinity in soils disturb ion balances and result in ROS production, oxidative stress and decreases in photosynthetic plant capability (Apel and Hirt, 2004; Wang et al., 2008). To prevent salt-induced damages in plants, these changes result in raises in the root/canopy ratio, and modifications in the leaf anatomy, xanthophyll cycle, photorespiration pathway, and water-water cycle (Acosta-Motos et al., 2017). Therefore, both stresses can impede plant growth and development by hindering nutrient absorption, inhibiting cell division and elongation, and disturbing the metabolic and photosynthetic system, that result in losses in crop yield (Li et al., 2013; Ullah et al., 2021).

Polyamines (PAs) are small aliphatic amines detected in all living organisms. In plants, the three major PAs include spermine (Spm), putrescine (Put) and spermidine (Spd), and are implicated in root elongation, leaf senescence, floral development, fruit ripening, programmed cell death, transcript expression and protein translation, and chromatin organization (Drolet et al., 1986). In addition, different genetic studies have revealed a key role for PAs in plant tolerance to different abiotic stresses. For example, a mutational approach to increase polyamine biosynthesis in *Oryza sativa* resulted in an enhanced oxidative stress tolerance by preventing the accumulation of ROS (Jang et al., 2012). Spd has been involved in modulating cell rescue and defense as well as antioxidant pathways in tomato seedlings subjected to heat stress (Sang et al., 2017), has been shown to increase the expression of transcripts encoding heat shock proteins to protect Arabidopsis plants from high temperatures (Sagor et al., 2012), and has been suggested to improve salinity tolerance of tomato plants (Zhang et al., 2015) and sorghum seedlings (Yin et al., 2016). In addition, Arabidopsis mutants deficient in Spm (*acl5/spms*) were hypersensitive to salinity and drought stress (Jang et al., 2012). Applications of exogenous PAs have been also reported to improve the tolerance of different plant species to several abiotic stresses, including salinity, high temperatures or drought stress (Mitsuya et al., 2009; Hu et al., 2012; Kamiab et al., 2014; Ahanger et al., 2019; Marco et al., 2019; Seifi et al., 2019; Acosta-Motos et al., 2020; Upadhyay et al., 2020; Sharma and Garg, 2021; ElSayed et al., 2022; Li et al., 2022). For example, Spm and Spd treatments have been correlated with an increased ROS scavenging, enhanced photosynthesis, improved plant growth, and decreased damaging impacts of salinity stress compared to non-treated plants (ElSayed et al., 2018; ElSayed et al., 2022). Moreover, applications of Put or Spm, or a mixture of them on wheat seedlings resulted in a positive modulation of drought responses by enhancing osmolyte accumulation, increasing free PA levels and regulating PA biosynthetic genes (Ebeed et al., 2017). Therefore, the role of the different PAs in promoting tolerance of several plant species to different abiotic stresses has been widely demonstrated. However, the effects of exogenous treatments of Spm on tomato responses to the combination of two important soil constraints, *i.e.*, salinity and the herbicide PQ have not been reported yet. In this work, the impact of the combination of salinity and PQ in the survival, growth, physiology, oxidative stress and the activity of different antioxidant enzymes in tomato Spm-treated and non-treated plants was evaluated. Our results show that plants treated with Spm improved their survival, growth, leaf damage, photosynthesis and photosystem (PSII) function when subjected to the combination of salinity and PQ, compared to stressed plants not treated with Spm. We further reveal that Spm function could be associated to reductions in oxidative stress induced by the impact of combined salinity and PQ, suggesting that Spm could alleviate the negative effects of this stress combination in tomato plants.

## 2 Materials and methods

### 2.1 Plant material and growth conditions

Montecarlo hybrid tomato seeds purchased from a commercial nursery (Seminis, Barásoain, Navarra, Spain) were used as plant material for exogenous Spm experiments. Tomato seeds were cultivated in seedling trays filled with peat moss, perlite and vermiculite (80:10:10). After germination, seedlings were transplanted to 10-cm diameter pots filled with peat moss and maintained under greenhouse conditions (70% relative humidity, 200 µmol photons m^-2^ s^-1^ light intensity, natural photoperiod day/night cycle with temperatures averaging 25.0°C and 18.0°C, respectively) and watered three times a week with half-strength Hoagland solution. Temperature and relative humidity were recorded regularly with a portable USB datalogger (OM-EL-WIN-USB, Omega, NJ, United States).

### 2.2 Stress treatments and experimental design

To study the effect of exogenous Spm in the tolerance of plants to different stresses and their combination, we treated 4-week-old tomato plants with Spm by watering with 0.5 L of distilled water containing 0.5 mM Spm (Sigma-Aldrich, St. Louis, MO, USA; Xu et al., 2020) once a day during a week, and control (CT) plants were watered in parallel with 0.5 L of distilled water (**Supplementary Figure 1**). After finishing Spm treatments, Spm-treated and not treated plants were subjected to four different conditions: CT, salinity (S, 150 mM NaCl), herbicide paraquat (PQ, 1.5 μM PQ), and the combination of salinity and paraquat (S+PQ, 150 mM NaCl + 1.5 μM PQ). Plants were watered three times a week for 15 days with each stressor described above (S, PQ and S+PQ) in a half-strength Hoagland solution. Therefore, eight experimental groups were designed (CT, CT+Spm, S, S+Spm, PQ, PQ+Spm, S+PQ, S+PQ+Spm), subjecting 5 plants to each stress treatment, and all experiments were repeated at least three times (**Supplementary Figure 1**). Once all stress treatments were completed, the number of healthy leaves (leaves with no symptoms of damage; Balfagón et al., 2019) and plant height were scored for all control and stressed plants, followed by sampling leaves in an intermediate position in the canopy in liquid N_2_. Samples were stored at -80°C until further use. For each analysis described below, at least three independent technical repeats per biological repeat and experimental group were performed.

### 2.3 Photosynthetic parameters and photosystem II efficiency

Photosynthetic rate, stomatal conductance, transpiration rate and PSII efficiency were measured simultaneously on plants subjected to the different stress treatments between 9:30 and 11:30 A.M. Leaf gas exchange parameters (photosynthetic rate, stomatal conductance and transpiration rate) were measured by using a LCpro^+^ portable infrared gas analyzer (LI-6800, LICOR, Lincoln NE, USA) under ambient CO_2_ and moisture. After instrument stabilization, six measurements were taken on three different mature fully expanded leaves in three replicate plants from each treatment. PSII efficiency was analyzed on the same leaves and plants using a portable fluorometer (FluorPen FP-MAX 100, Photon Systems Instruments, Czech Republic).

### 2.4 Malondialdehyde analysis

Approximately 200 mg of ground frozen leaf tissue was homogenized in 2 mL of 80% ethanol (Panreac) by sonication for 30 min. Homogenates were then centrifuged at 12000 g for 10 min and different aliquots of the supernatant were mixed either with 20% trichloroacetic acid or with a mixture of 20% trichloroacetic acid and 0.5% thiobarbituric acid. Both mixtures were incubated in a water bath at 90°C for 1 h and, after cooling down, samples were centrifuged at 5000 g for 5 min at 4°C. The absorbance of the supernatant was read at 440, 534 and 600 nm against a blank, and malondialdehyde (MDA) concentration was calculated as described in Zandalinas et al. (2017).

### 2.5 H_2_O_2_ accumulation

H_2_O_2_ accumulation in leaves was measured by using a commercial kit (Amplex Red hydrogen peroxide-peroxidase assay, Molecular Probes/Invitrogen, Eugene, OR, USA) with few modifications. Briefly, 500 μL of 50 mM sodium phosphate buffer at pH 7.4, containing 50 μM of Amplex Red reagent and 0.05 U mL^-1^ of horseradish peroxidase, was added to approximately 40 mg of frozen leaf tissue and incubated for 30 min at room temperature in darkness. Then, samples were centrifuged at 12000 g for 12 min at 4°C and 50 μL of supernatants were transferred into new opaque tubes. Absorbance at 560 nm was measured by using a NanoDrop Spectrophotometer (Thermo Fisher Scientific, Wilmington, DE, USA). The concentration of H_2_O_2_ in each sample was determined from a standard curve consisting of 0, 0.5, 1, 3, 6, and 9 μM H_2_O_2_. After absorbance measurements, H_2_O_2_ accumulation per mg of fresh weight was calculated.

### 2.6 RNA isolation, primer design and RT-qPCR

RNA was extracted from frozen leaf tissue using an RNeasy Mini kit (Qiagen, Hilden, Germany) following the manufacturer’s instructions. Total RNA concentration and purity were determined using a Nanodrop 2000 spectrophotometer (Thermo Scientific, Wilmington, DE) from the ratio of absorbance readings at 260 and 280 nm. Reverse transcription was performed from 1 μg of total RNA using Primer script RT reagent with oligo(dT) primer (Takara Bio Inc., Kusatsu, Japan). The specific primers used for the amplification of each gene are included in **Supplementary Table 1**. Primer pairs used for the tomato amplification were designed using free surface Primer3Plus (https://www.primer3plus.com) and NCBI database. Relative expression analysis by RT-qPCR were performed in a StepOne Real-Time PCR system (Applied Biosystems, Foster City, CA, United States). The reaction mixture contained 1 μL of cDNA, 5 μL of SYBRGreen (Applied Biosystems) and 1 μM of each gene-specific primer pair in a final volume of 10 μL. The thermal profile used to analyze the relative gene expression consisted of 10 min at 95°C for pre-incubation, followed by 40 cycles of 10 s at 95°C for denaturation, 10 s at 60°C for annealing and 20 s at 72°C for the extension. Amplicon specificity of the PCR reaction was evaluated by the presence of a single peak in the dissociation curve after the amplification steps. The expression levels of all genes were normalized against the expression of two endogenous control genes (actin [Solyc03g078400] and GAPDH [Solyc05g014470]) based on previous housekeeping selection for tomato (Mascia et al., 2010) and the relative expression was calculated by using REST (Pfaffl et al., 2002). For all genes studied, the reference for S, PQ and S+PQ samples was the expression value obtained for the CT conditions, whereas the reference for S+Spm, PQ+Spm and S+PQ+Spm was the expression value obtained for the CT+Spm conditions. Reference conditions were set as 1. Three technical replicates were analyzed on each biological replicate.

### 2.7 Antioxidant enzyme activities

To determine the superoxide dismutase (SOD), catalase (CAT) and glutathione reductase (GR) activities, about 100 mg of frozen leaf tissue was extracted in 2 mL of phosphate buffer at pH 7 in a ball mill (MillMix20, Domel, Železniki, Slovenija). After centrifugation at 14000 g at 4°C for 10 min, supernatants were recovered. Different buffers were used for each enzyme extraction: for SOD, 50 mM phosphate buffer (pH 6.8) with 1.33 mM diethyl-diamino-pentaacetic acid; for CAT and GR, 50 mM phosphate buffer (pH 6.8 and pH 7.5, respectively). The SOD activity was determined following the ${\text{O}}_{2}^{\;-}$ -induced reduction of nitroblue tetrazolium using the xanthine–xanthine oxidase system. CAT activity was determined using the H_2_O_2_-dependent reduction of titanium chloride. The GR activity was studied following the increase in the absorbance at 412 nm during 2 min as result of the production of the adduct DTNB-GSH after GSSG reduction. The reaction was initiated by adding 10 µL of enzyme extract and the increment in absorbance was recorded during 2 min at 265 nm. Enzyme activity was expressed as enzyme unit (U) per mg of protein. Total protein amount of each extract was determined following the method of Bradford (1976) using Bovine Serum Albumin (BSA) as a protein standard. Further details on enzyme assays are provided in Hossain et al. (2009).

### 2.8 Total antioxidant enzyme activity inhibition

To determine the total antioxidant enzyme activity inhibition (Lado et al., 2016), 50 mg of frozen leaf tissue was extracted in 1 mL of MeOH 80% in a ball mill (MillMix20, Domel, Železniki, Slovenija). After centrifugation at 14000 g at 4°C for 10 min, supernatants were recovered. A buffer containing 738 µM ABTS (Sigma-Aldrich, St. Louis, MO, USA) and 245 µM K_2_S_2_O_8_ (Sigma-Aldrich, St. Louis, MO, USA) was prepared and incubated at room temperature in darkness. Then, a blank with the previous solution buffer was adjusted to an absorbance of 700 nm, and 10 µL of each extract was added to 1 mL of buffer mix. Measurements at 734 nm were recorded at different times (0, 0.5, 1 and 2 mins). Antioxidant activities were determined using ABTS reduction in potassium persulfate and were calculated as U per mg of protein contained in each extract.

### 2.9 Statistical analysis

Results are presented as the mean ± SD. Statistical analyses were performed by two-tailed Student’s *t-*test. * denotes statistical significance at P< 0.05 with respect to CT and * means statistical significance at P< 0.05 between Spm-treated and non-treated plants exposed to the same stress condition.

## 3 Results

### 3.1 Survival, healthy leaves and growth of Spm-treated and non-treated tomato plants subjected to combined salinity and paraquat

**Figure 1:**
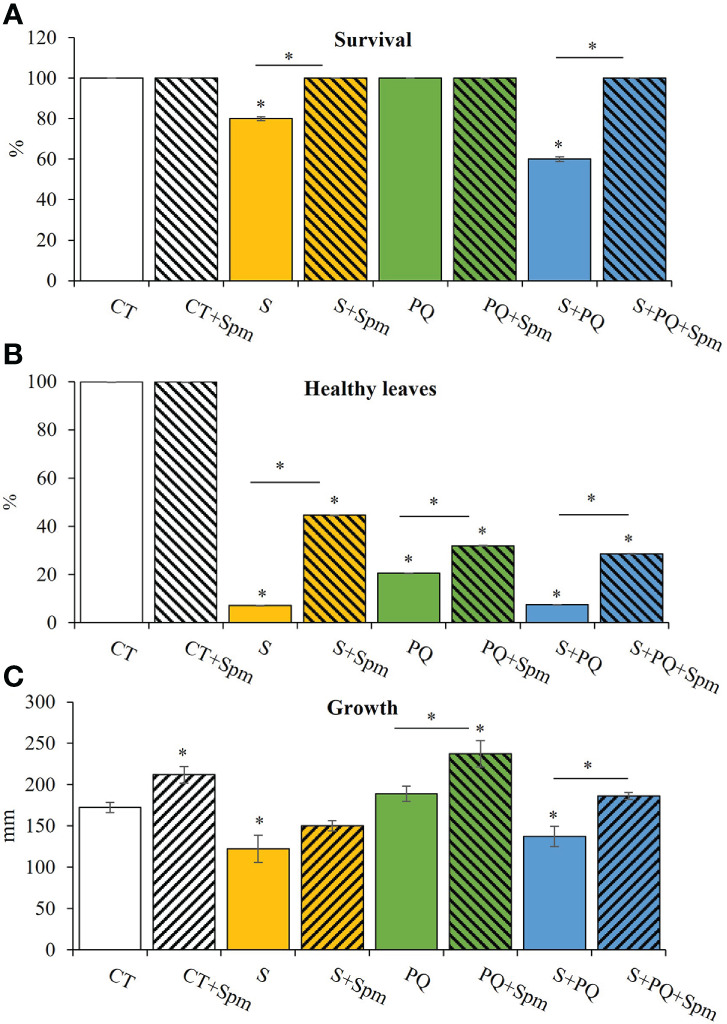
Involvement of Spm treatment in the survival, percentage of healthy leaves and growth of tomato plants subjected to salinity, paraquat and the combination of salinity and paraquat. **(A)** Survival, **(B)** percentage of healthy leaves, and **(C)** growth of non-treated and Spm-treated tomato plants subjected to the different stresses. All experiments were repeated at least three times with similar results. Values indicate the mean ± standard deviation. * refers to statistical significance at P< 0.05 with respect to CT and * means statistical significance at P< 0.05 between Spm-treated and non-treated plants exposed to the same stress condition. CT, control; S, salinity; Spm, spermine; PQ, paraquat.

To study the survival and growth of Spm-treated and non-treated tomato plants in response to S, PQ and their combination (S+PQ), plants were subjected to 150 mM NaCl, 1.5 µM PQ or 150 mM NaCl and 1.5 µM PQ, respectively, for 15 days (**Supplementary Figure 1**). As shown in **Figure 1A**, survival of tomato plants not treated with Spm significantly decreased in response to S (around 80%) and more markedly in plants subjected to S+PQ (around 60%), whereas all plants survived when subjected to PQ. Interestingly, all Spm-treated plants subjected to the different stresses survived at the end of the different stress treatments (**Figure 1A**). Similarly, plants treated with Spm and subjected to S, PQ and S+PQ showed more healthy leaves and less visible toxicity symptoms such as chlorosis and necrosis (Balfagón et al., 2019), compared to plants not treated with Spm and subjected to the different stresses (**Figure 1B**). In addition, Spm treatment significantly increased tomato growth under control or stress conditions of PQ and S+PQ (**Figure 1C**).

### 3.2 Photosynthetic and gas exchange parameters of Spm-treated and non-treated tomato plants subjected to combined salinity and paraquat

**Figure 2:**
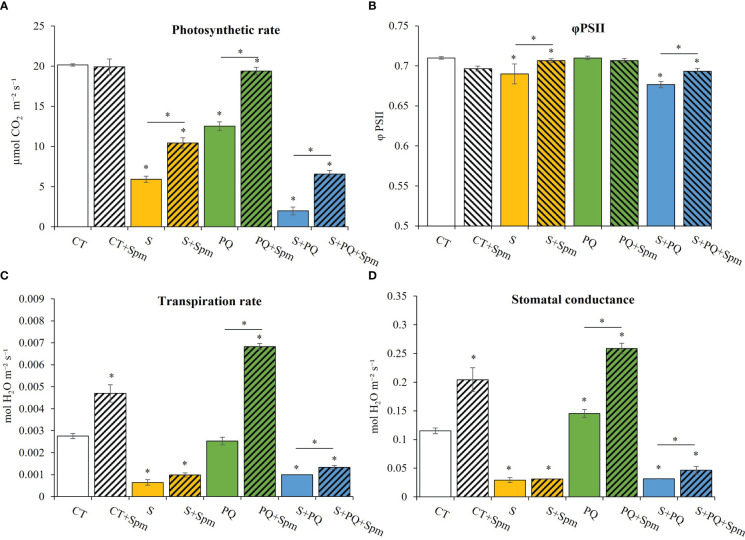
Involvement of Spm treatment in the photosynthetic rate, PSII function, and gas exchange parameters of tomato plants subjected to salinity, paraquat and the combination of salinity and paraquat. **(A)** photosynthetic rate, **(B)** PSII efficiency, **(C)** transpiration rate, and **(D)** stomatal conductance of non-treated and Spm-treated tomato plants subjected to the different stresses. All experiments were repeated at least three times with similar results. Values indicate the mean ± standard deviation. * refers to statistical significance at P< 0.05 with respect to CT and * means statistical significance at P< 0.05 between Spm-treated and non-treated plants exposed to the same stress condition. CT, control; S, salinity; Spm, spermine; PQ, paraquat; PSII, photosystem II.

To determine whether Spm could affect the photosynthesis and different gas exchange parameters of control plants, and plants subjected to S, PQ and S+PQ, photosynthetic rate, PSII efficiency, transpiration rate and stomatal conductance were monitored in plants treated and non-treated with Spm (**Figure 2**). Photosynthetic rate levels significantly decreased in response to all individual and combined stresses in non-treated plants compared to CT, reaching the lowest value when plants were subjected to S+PQ (**Figure 2A**). Interestingly, exogenous Spm application enhanced the photosynthetic rate under each individual and combined stress. In this sense, Spm-treated plants subjected to S, PQ and S+PQ increased by 6.78%, 7.67% and 33.3%, respectively, their photosynthetic rate. PSII efficiency was negatively affected by S and S+PQ, and Spm treatment increased these values by 13.21% and 8.29%, respectively (**Figure 2B**). Not surprisingly, stomatal conductance of plants treated and non-treated with Spm from the different stresses similarly corresponded to the transpiration rates measured in these plants (**Figures 2C, D**). Therefore, whereas plants subjected to S and S+PQ, and non-treated with Spm reduced transpiration rate and stomatal conductance levels, PQ did not change or slightly increased these values. Interestingly, Spm treatment increased both gas exchange parameters in CT plants as well as in plants subjected to PQ and S+PQ with respect to the corresponding non-treated stressed plants (**Figures 2C, D**). Taking together, Spm could be involved in promoting stomata aperture in response to PQ and S+PQ, as well as under control conditions.

### 3.3 Oxidative stress and antioxidant response of Spm-treated and non-treated tomato plants subjected to combined salinity and paraquat

**Figure 3:**
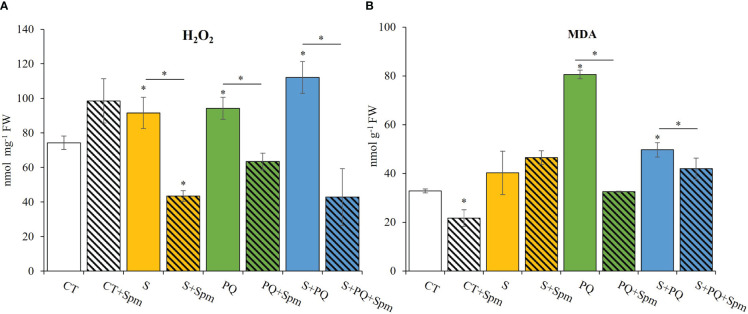
Involvement of Spm treatment in H_2_O_2_ and MDA accumulation in tomato plants subjected to salinity, paraquat and the combination of salinity and paraquat. **(A)** H_2_O_2_ accumulation, and **(B)** MDA levels of non-treated and Spm-treated tomato plants subjected to the different stresses. All experiments were repeated at least three times with similar results. Values indicate the mean ± standard deviation. * refers to statistical significance at P< 0.05 with respect to CT and * means statistical significance at P< 0.05 between Spm-treated and non-treated plants exposed to the same stress condition. CT, control; FW, fresh weight; MDA, malondialdehyde; S, salinity; Spm, spermine; PQ, paraquat.

**Figure 4:**
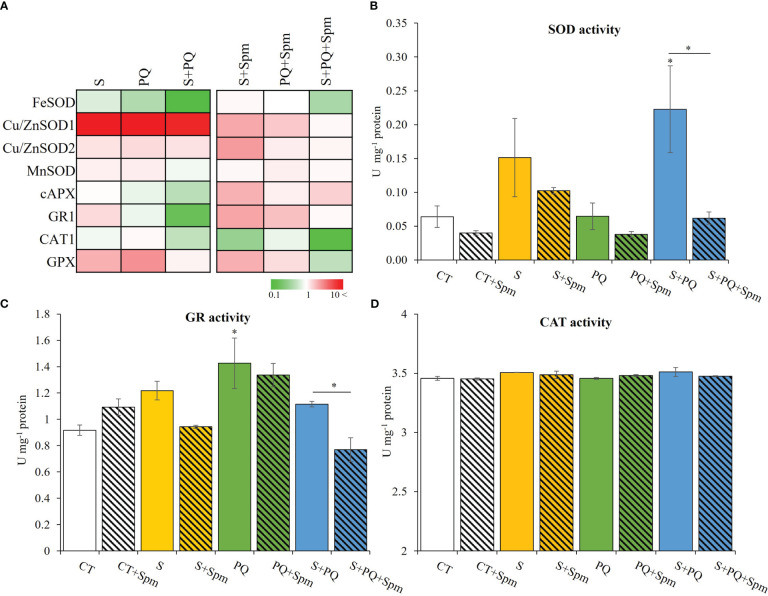
Involvement of Spm treatment in the expression of transcripts encoding antioxidant enzymes and their activities in tomato plants subjected to salinity, paraquat and the combination of salinity and paraquat. **(A)** Relative expression of transcripts encoding the antioxidant enzymes FeSOD, Cu/ZnSOD1, Cu/ZnSOD2, MnSOD, cAPX, GR1, CAT1 and GPX. **(B–D)** Activities of the antioxidant enzymes SOD **(B)**, GR **(C)** and CAT **(D)** in non-treated and Spm-treated tomato plants subjected to the different stresses. All experiments were repeated at least three times with similar results. Values indicate the mean ± standard deviation. * refers to statistical significance at P< 0.05 with respect to CT and * means statistical significance at P< 0.05 between Spm-treated and non-treated plants exposed to the same stress condition. cAPX, cytosolic ascorbate peroxidase; CAT, catalase; CT, control; GPX, glutathione peroxidase; GR, glutathione reductase; S, salinity; SOD, superoxide dismutase; Spm, spermine; PQ, paraquat.

To gain a better understanding of the impact of each stress condition and the Spm-associated changes on the oxidative stress, H_2_O_2_ levels, MDA content, RT-qPCR analyses of transcripts involved in detoxification pathways, as well as different antioxidant enzyme (SOD, GR and CAT) activities were studied (**Figures 3**, **4**; **Supplementary Figure 2**). Plants not treated with Spm and subjected to the different stresses significantly increased their leaf H_2_O_2_ levels compared to CT, whereas Spm treatment reduced the amount of H_2_O_2_ accumulated in plants subjected to S, PQ and S+PQ with respect to the corresponding stressed plants not treated with Spm (**Figure 3A**). In addition, the degree of lipid peroxidation in tomato plants was studied by monitoring changes in MDA levels (Taulavuori et al., 2001). As shown in **Figure 3B**, the level of lipid peroxidation decreased by about 56% and 12% in plants subjected to PQ+Spm and S+PQ+Spm, with respect to PQ and S+PQ, respectively.

The activation of detoxification pathways in Spm-treated and non-treated tomato plants subjected to the different stress conditions was evaluated by assessing the expression of FeSOD, Cu/ZnSOD1, Cu/ZnSOD2, MnSOD, CAT1, GPX, cAPX and GR transcripts as well as enzymatic activities of SOD, GR and CAT (**Figure 4**; **Supplementary Figure 2**). In general, Spm treatment modified the expression of all the transcripts tested involved in detoxification processes compared to that observed in stressed plants not treated with Spm (**Figure 4A**; **Supplementary Figure 2**). The expression of Cu/ZnSOD1, Cu/ZnSOD2, CAT1 and GPX was, in general, attenuated due to the effect of Spm treatment in plants subjected to the different stresses, whereas the expression of cAPX and GR1 was enhanced as a result of exogenous Spm application compared to that shown in stressed plants not treated by Spm (**Figure 4A**; **Supplementary Figure 2**). To further analyze the role of Spm in ROS scavenging processes, the activities of SOD, GR and CAT were evaluated (**Figures 4B–D**). S or S+Spm did not alter any enzymatic activity, and PQ increased GR activity. Under S+PQ conditions, a significant decrease of SOD and GR enzymatic activities in Spm-treated plants by 21.21% and 66.67%, respectively, was observed compared to non-treated plants subjected to S+PQ. On the contrary, CAT activities did not show any alteration in response to stress in both Spm-treated and non-treated plants (**Figure 4D**).

Additionally, to determine the total antioxidant capacity of plants, analysis of the inhibition of total antioxidant activities was performed (**Table 1**). Spm treatment enhanced the total antioxidant capacity of plants under CT conditions. Moreover, PQ and S+PQ resulted in increased levels of total antioxidant activity compared to CT values. By contrast, values of total inhibition of antioxidant activities decreased in plants subjected to S+PQ+Spm, compared to S+PQ (**Table 1**).

**Table 1:** Involvement of Spm treatment in the total antioxidant enzymatic inhibition of tomato plants subjected to salinity, paraquat and the combination of salinity and paraquat.

	Average inhibition antioxidant enzymatic activity (% compared to time 0)		
Treatments	0.5 min	1 min	2 min
CT	68.37 ± 10.70	71.59 ± 9.72	74.22 ± 8.77
CT+Spm	81.97 ± 2.48*	86.22 ± 0.72*	89.46 ± 2.37*
S	56.99 ± 11.73	62.96 ± 10.48	68.79 ± 10.93
S+Spm	64.58 ± 4.34	74.89 ± 1.53	83.28 ± 6.54*
PQ	127.93 ± 3.65*	135.86 ± 5.52*	142.21 ± 5.01*
PQ+Spm	126.11 ± 9.54*	130.30 ± 10.94*	137.69 ± 10.46*
S+PQ	122.46 ± 4.61*	130.75 ± 3.02*	137.55 ± 4.40*
S+PQ+Spm	105.14 ± 3.04*†	112.30 ± 0.62*†	121.02 ± 2.33*†

Values indicate the mean ± standard error. * refers to statistical significance at P< 0.05 with respect to CT and † means statistical significance at P< 0.05 between Spm-treated and non-treated plants exposed to the same stress condition. CT, control; PQ, paraquat; S, salinity; Spm, spermine.

## 4 Discussion

The critical influence of PAs in protecting plant cells from several abiotic stresses has been extensively demonstrated in different plant species (reviewed in Zandalinas et al., 2022). However, the potential role of the different plant PAs in regulating plant responses to different abiotic stress combinations is not fully understood (Zandalinas et al., 2022). For example, previous reports suggested a key role for Spm in conferring tolerance of trifoliate orange seedlings exposed to the combination of heat and drought (Fu et al., 2014). In addition, modulation of PA biosynthesis was correlated with a better protection of tobacco plants against the combination of heat and drought (Cvikrová et al., 2013). On the contrary, other stress combinations including high light and heat stress, induced the repression of PAs such as Put, suggesting that Put may have a marginal effect on plant acclimation to this stress combination (Balfagón et al., 2022). In this work, we expanded the knowledge about the action of Spm in tomato plant tolerance to the combination of another important abiotic stress combination (*i.e*., salinity and the herbicide PQ). Our results revealed that application of exogenous Spm enhanced survival, growth, photosynthetic rate and PSII function of tomato plants subjected to the combination of S and PQ (S+PQ) compared with plants subjected to S+PQ and not treated with Spm, while decreasing the leaf damage associated with the different stresses (**Figures 1**, **2A, B**). In agreement with previous reports proposing Spm as a stomatal regulator (*e.g*., Hassan et al., 2018; Berahim et al., 2021), our results indicate that Spm treatments induced stomatal aperture and enhanced transpiration in tomato plants under PQ and S+PQ (**Figures 2C, D**). Therefore, the Spm-induced improvement in photosynthetic rate of tomato plants subjected to PQ and S+PQ (**Figure 2A**) could be partially attributed to the increased stomatal aperture associated with Spm treatment (**Figure 2D**), resulting in a potential increment of internal CO_2_ concentration in plant cells. These results suggest that Spm could be involved in modulating stomata aperture resulting in contrasting outcomes depending on the plant species and/or the stress or stress combination involved. Moreover, the improvement in the photosynthetic rate observed in response to stresses involving S (S and S+PQ; **Figure 2A**) in plants treated with Spm could be attributed to the Spm-induced increase in PSII function under these stress conditions (**Figure 2B**). Further studies are needed to determine the specific molecular mechanisms of Spm in controlling stomata changes as well improving PSII activity under these stress situations.

It has been previously reported that S and PQ stresses applied individually result in oxidative damage to plants (Wang et al., 2008; Hawkes, 2014), and that an increase in antioxidant capacities of cells is a key strategy against stress-induced oxidative damage (Yadav et al., 2011). Our results indicate that Spm treatments significantly reduced the amount of H_2_O_2_ accumulated in response to S, PQ and S+PQ (**Figure 3A**), as well as stressed-induced MDA levels in response to PQ and S+PQ (**Figure 3B**). Therefore, Spm played a key role in alleviating a potential stress-induced oxidative damage as well as reduced lipid peroxidation processes that could occur in plants subjected to PQ or S+PQ. In agreement with our data, Li et al. (2022) reported a positive correlation between Spm treatment and improvements in photosynthetic performance, antioxidant capacity and redox homeostasis in creeping bentgrass subjected to salinity.

The exact mechanism by which PAs confer protection to different stresses remains unclear (Marco et al., 2011). Their role on plant tolerance to stress has been associated with their capacity to regulate transcription and translation, maintain membrane stability, and modulate antioxidant processes (reviewed in Liu et al., 2015). The lower oxidative stress of Spm-treated tomato plants subjected to S+PQ (assessed by reduced content in H_2_O_2_ and MDA; **Figure 3**), could result in alterations in the expression of transcripts encoding antioxidant enzymes, in a reduction of their activities (**Figure 4**), as well as in a lower total antioxidant capacity of plant cells under stress (**Table 1**). In addition, the Spm-induced decline in the oxidative damage associated to S+PQ could be also correlated to less PSII damage (**Figure 2B**) and, in turn, in enhanced photosynthetic rates (**Figure 2A**). Possible mechanisms by which Spm could trigger ROS reduction include its role in inhibiting the auto-oxidation of metals that leads to impairment of the electron supply for ROS generation (Shi et al., 2010), or its function as direct antioxidants and ROS scavengers as proposed previously (Liu et al., 2015) and suggested in our data by the increment of the total antioxidant capacity of CT plants treated with Spm (**Table 1**). Further studies are needed to decipher the specific mechanism utilized by Spm to reduce the oxidative pressure of plant cells subjected to S+PQ.

Taking together, our findings show that tomato plants treated with Spm improved their survival, growth, leaf damage, photosynthesis and PSII function in response to S+PQ. In addition, our results indicate that Spm is involved in ameliorating the negative effects of S+PQ by reducing the stress-associated oxidative pressure and suggest that Spm could play a key role in tomato plant responses to combined stress. Further studies are of course required in order to determine the exact molecular mechanism by which Spm and the other main polyamines (putrescine and spermidine) may function under this stress combination.

## Data availability statement

The original contributions presented in the study are included in the article/**Supplementary Material**. Further inquiries can be directed to the corresponding authors.

## Author contributions

LP and CS-M performed the research; LP and SZ designed the research, prepared figures, and wrote the manuscript with contributions of AG-C and ML-C. SZ and AG-C supervised the research, provided laboratory infrastructure and funding. All authors contributed to the article and approved the submitted version.
