# Supplementary material for: Exogenous spermine alleviates the negative effects of combined salinity and paraquat in tomato plants by decreasing stress-induced oxidative damage
Source: Front Plant Sci. 2023 May 9;14:1193207. doi: 10.3389/fpls.2023.1193207 (PMC10203479; doi:10.3389/fpls.2023.1193207)

Supplementary Material

Exogenous spermine alleviates the negative effects of combined salinity and paraquat in tomato plants by decreasing stress-induced oxidative damage

Lidia S. Pascual, María F. López-Climent, Clara Segarra-Medina, Aurelio Gómez-Cadenas*, Sara I. Zandalinas*

***Correspondence:** Sara I. Zandalinas, sizquier@uji.es; Aurelio Gómez-Cadenas, aurelio.gomez@uji.es

1 Supplementary Figures and Tables

1.1 Supplementary Tables

Supplementary Table 1. Transcript-specific primers used for relative expression analysis by RT-qPCR**.** Abbreviations used: cAPX, cytoplasmatic ascorbate peroxidase; CAT, catalase; GPX, glutathione peroxidase; GR, glutathione reductase; SOD, superoxide dismutase.

Gene	Accession number	Forward	Reverse
SlActin	Solyc03g078400	GGTATCGTCCTGGACTCTGGTG	GGGAAGGGCGTAACCTTCA
SlcAPX	Solyc06g005150	TCTGAATTGGGATTTGCTGA	CGTCTAACGTAGCTGCCAAA
SlGAPDH	Solyc05g014470	ACAACTTAACGGCAAATTGACTGG	TTACCCTCTGATTCCTCCTTGATTG
SlCAT1	Solyc12g094620	TGATCGCGAGAAGATACCTG	CTTCCACGTTCATGGACAAC
SlGPX	Solyc08g080940	ACGGAGCAAGCGACAATTGACAAC	CGATTGATTCACCGCAAAGCTCGT
SlGR1	Solyc08g074850	TTGGTGGAACGTGTGTTCTT	TCTCATTCACTTCCCATCCA
SlFeSOD	Solyc06g048410	GGATGCTTTGGAGCCTCATA	GAAGGGGAGCACCATTGTTA
SlMnSOD	Solyc02g082590	GAATAACCTTGCCCCTGTCA	AGCACCTTCTGCGTTCATCT
SlCu/ZnSOD1	Solyc08g079830.2	GGCCAATCTTTGACCCTTTA	AGTCCAGGAGCAAGTCCAGT
SlCu/ZnSOD2	Solyc08g079830.2	AGTGGCACCATCCTCTTCAC	GGACATGGAAGCCATGAAGT

1.2 Supplementary Figures

Supplementary Figure 1. The experimental design used for the study of the involvement of Spm treatment in tomato responses to salinity, paraquat and the combination of salinity and paraquat. One week after transplanting plants, half of them were watered with 0.5 mM Spm for 7 days (Spm-treated plants), and the other half of the plants were watered with distilled water (non-treated plants). After Spm treatments, plants with and without exogenous Spm application were watered with half strength Hoagland solution containing 150 mM NaCl, and/or 1.5 µM PQ. All experiments were repeated three times with at least five plants per stress treatment. Abbreviations: CT, control; PQ: paraquat; S, salinity; Spm: spermine.

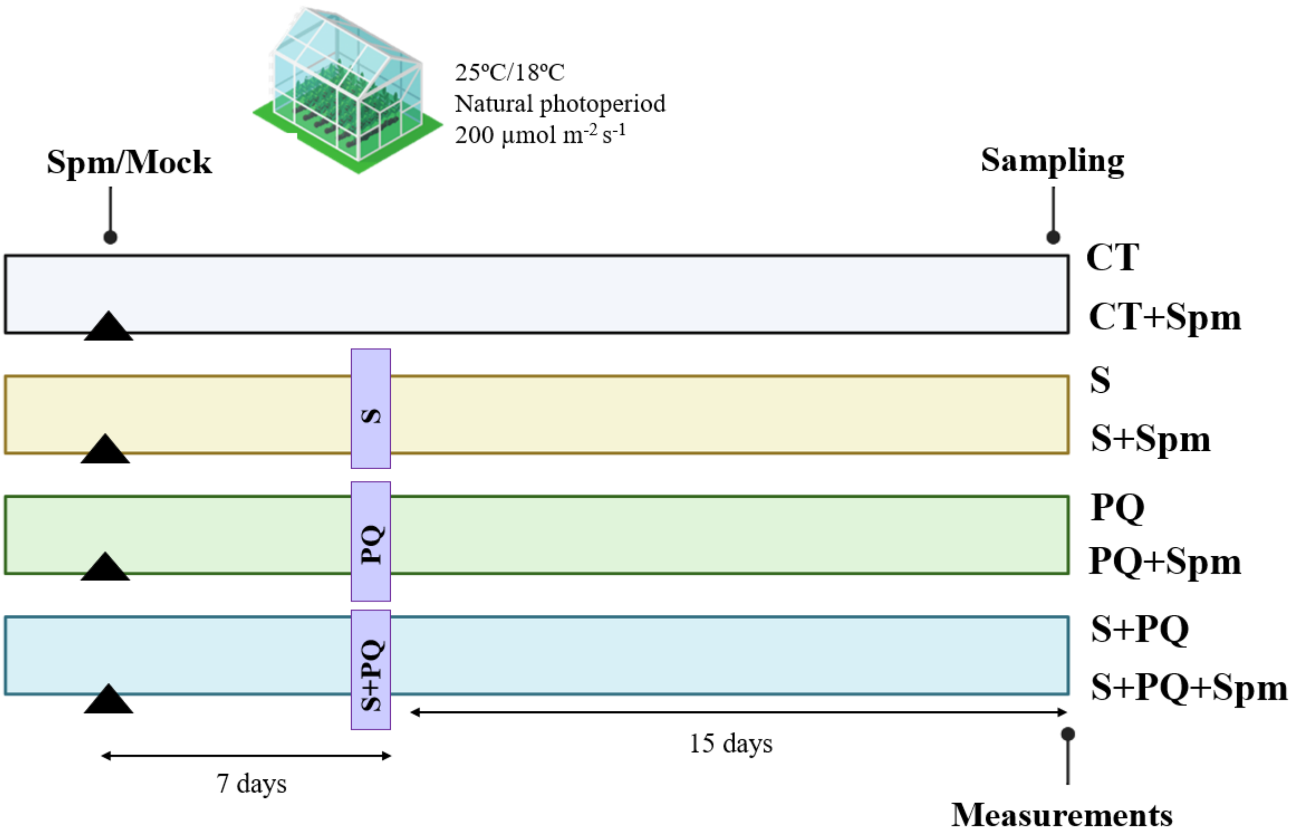

Supplementary Figure 2. Bar graphs showing the involvement of Spm treatment in the relative expression of transcripts encoding the antioxidant enzymes FeSOD, Cu/ZnSOD1, Cu/ZnSOD2, MnSOD, cAPX, GR1, CAT1 and GPX in tomato plants subjected to salinity, paraquat and the combination of salinity and paraquat. Values indicate the mean ± standard error. For all genes studied, the reference for S, PQ and S+PQ samples was the expression value obtained for the CT conditions, whereas the reference for S+Spm, PQ+Spm and S+PQ+Spm was the expression value obtained for the CT+Spm conditions. Reference conditions were set as 1. * refers to statistical significance at $P < 0.05$ with respect to CT (S, PQ, S+PQ) or to CT+Spm (S+Spm, PQ+Spm, S+PQ+Spm). Abbreviations used: cAPX, cytoplasmatic ascorbate peroxidase; CAT, catalase; CT, control; GPX, glutathione peroxidase; GR, glutathione reductase; PQ: paraquat; S, salinity; SOD, superoxide dismutase; Spm: spermine.

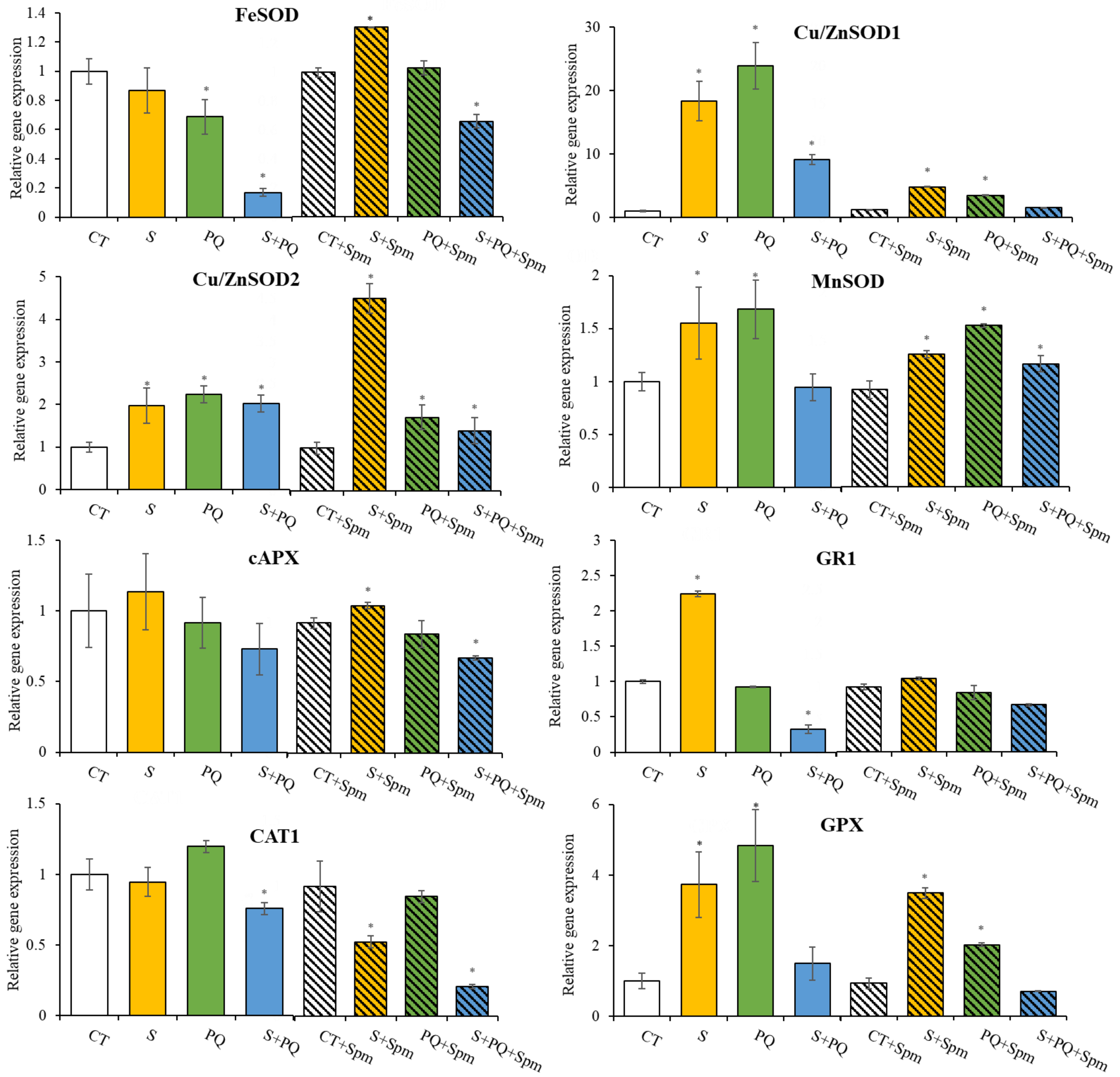

Supplement: Supplementary file 1 [file DataSheet_1.pdf]
